# Supplementary material for: Transcriptomic Insights into Mechanisms of Early Seed Maturation in the Garden Pea (Pisum sativum L.)
Source: Cells. 2020 Mar 23;9(3):779. doi: 10.3390/cells9030779 (PMC7140803; doi:10.3390/cells9030779)

Sprint−2(10) vs Zhewa

Global Summary

%DE = 0.89

genes with fdr < 0.2 = 1846 (955 + / 891 −)

genes with fdr < 0.1 = 1790 (935 + / 855 −)

genes with fdr < 0.05 = 1611 (847 + / 764 −)

genes with fdr < 0.01 = 1332 (712 + / 620 −)

genes in genesets = 0

<FC> = 0

<t−score> = −50.7

<p−value> = 0

<fdr> = 0.11

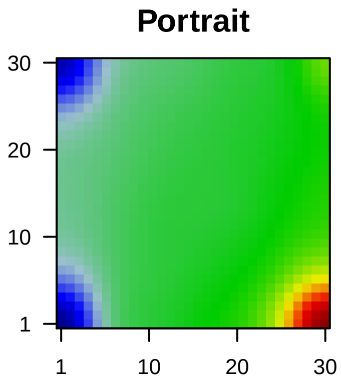

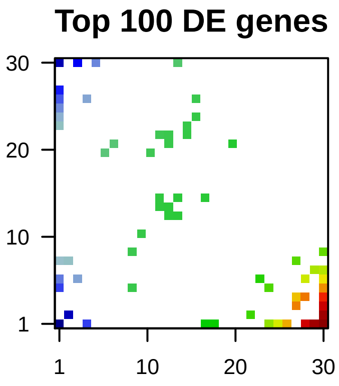

Global Genelist

Rank	ID	log(FC)	p−value	fdr	Metagene	Description
1	TRINITY_DN1	−17.89	6e−15	1e−11	1 x 1	
2	TRINITY_DN4	−14.15	5e−14	1e−07	1 x 30	
3	comp52436_c	−0.05	5e−10	1e−07	20 x 21	
4	TRINITY_DN1	−0.04	1e−09	1e−07	9 x 9	
5	TRINITY_DN1	−0.01	3e−09	1e−07	15 x 22	
6	TRINITY_DN1	−0.07	3e−09	1e−07	13 x 21	
7	TRINITY_DN1	0.56	3e−09	1e−07	27 x 4	
8	comp41910_c	−0.04	3e−09	1e−07	9 x 5	
9	TRINITY_DN2	−1.01	4e−09	1e−07	1 x 30	
10	TRINITY_DN1	−0.04	4e−09	3e−07	11 x 20	
11	TRINITY_DN7	−0.03	8e−09	3e−07	10 x 11	
12	TRINITY_DN1	−1.3	1e−08	3e−07	1 x 30	
13	TRINITY_DN5	−0.01	1e−08	3e−07	12 x 15	
14	TRINITY_DN2	−2.3	1e−08	3e−07	1 x 1	
15	TRINITY_DN1	−1.57	1e−08	3e−07	1 x 1	
16	TRINITY_DN4	0.43	1e−08	3e−07	28 x 6	
17	TRINITY_DN1	−0.01	2e−08	3e−07	13 x 14	
18	TRINITY_DN2	−0.17	2e−08	3e−07	6 x 20	
19	TRINITY_DN8	−0.34	2e−08	3e−07	1 x 23	
20	TRINITY_DN6	1.53	2e−08	3e−07	30 x 1	

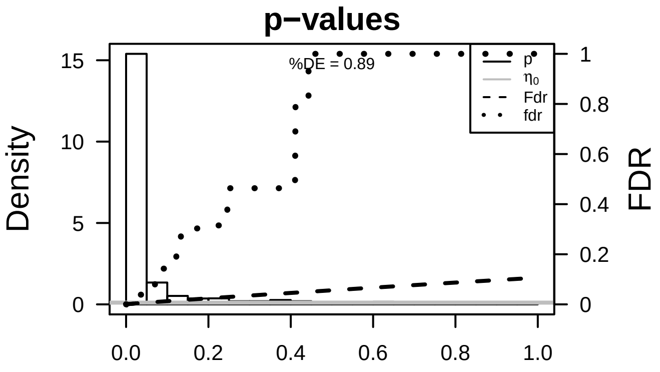

Supplement: Supplementary file 1 [file cells-09-00779-s001.zip › Supplementary materials/FileS4.zip!/FileS3/TFs - Results/Summary Sheets - Differences/Reports/Sprint.2.10..vs.Zhewan.1.10..pdf]
